# Sundry Paragraphs Too Numerous for This Limited Space—Read Them

**Published:** 1886-08

**Authors:** 


### Editorial Notes.

*Appointments T. S. M. A.*—The President of the Texas State Medical Association has appointed Drs. R. M. Swearingen and J. W. McLaughlin on the Publication Committee in place of Dr. Q. C. Smith, declined to serve, and Dr. W. J. Burt, deceased. The Transactions will appear much later than was anticipated, for reasons which it would not do to publish--just yet.

---

*A Call* has been issued to the Medical profession of the South and West to meet with the Mississippi Valley Medical Association at Crab Orchard, Kentucky, in July 1887, to form a Southern and Western Medical Association—a kind of cut loose from our Eastern brethren.

---

*Poor Baby.*—A writer in an exchange says: "If the baby does not thrive on fresh milk it should be boiled."

Another one, speaking of a new nursing-bottle, says: "When the baby has done sucking it should be unscrewed and hung up."

In the forthcoming Transactions of the Texas State Medical Association there will be an article in which the author, speaking of a sick child says: "The stomach was very irritable and it kept twiching its mouth."

---

*Dog Days* seem to take all the life out of most people. Our exchanges for August are mostly as tame as stale cider.

---

*P. M. B. A.*—Members of the Physician's Mutual Benefit Association of Texas, who have failed to respond to the asessment for the benefit of the heirs of Brother Dr. D. Sayres, which assessment was mailed July 10th, are again called on to send forward their $1. Failure to do so by the end of August will forfeit membership; and the names of all such as fail to make good their pledge, will be erased from the roll.

F. E. Daniel, M. D.
Sec. and Treasurer.

SURGEON GENERAL BAXTER, U. S. A.—That is the way it will read very shortly, unless we greatly mistake; for General Murray, having reached the age of 62, will be retired, according to army regulations, and Col. J. H. Baxter, who is now, and has been, during Gen. Murray's administration, the First Assistant Surgeon General, is the ranking Colonel of the six Assistants Surgeon General on the roster. Moreover, Dr. Baxter has every claim to the position that attaches to long and faithful service, intelligent and satisfactory discharge of duty,—competency—capacity, a thorough acquaintance with the responsible duties of this the highest office in the medical department of the army. It is thought, too, by many, that injustice was done him by the late president Arthur in not making him Surgeon General at the time Dr. Murray was appointed, his friends claiming that he even out ranks Gen. Murray. There is a kink somewhere in the date of commissions, on which a doubt hangs—and Dr. Baxter did not get the benefit of the doubt at that time; but now, there is *no doubt* as to his seniority; and we should not like to hear any man say there is doubt on any other score, as to his fitness to discharge the duties with ability, or to grace and adorn the office with his many admirable qualities.

---

A NEW BONE.—In the *Brief* for July is an account by Dr. H. C. Bennett, of New Salem, Texas, of "a twin-headed baby," which he was compelled to remove piecemeal recently. On page 296, the Doctor says: "We then took off the other leg, together with the asinnomate bone."

If the Doctor had preserved this rare specimen, and exhibited it along with the "twin-headed baby," it would have immortalized the baby—the only one ever known to possess an "asinnomate bone."

---

DOT YOUR EYES—As an illustration of the want of accuracy in writing, observed in many distinguished men, we mention the receipt of a letter from a well known physician, in which he said: "I have been expecting to hear from you daily." We replied that we had no occasion to write daily, nor had we the time or inclination to do so. Of course he meant to say he had "daily been expecting to hear," etc.

---

*Stay-at-home Young Lady to Traveled Young Lady*—"I suppose you spent a week or more in Rome; tell me about the sights you saw."

*T. Y. L.*—Rome? Rome? Let me see! Ah, no! we passed through there in the night."

---

TEXAS SURGERY—LAPAROTOMY FOR INTESTINAL WOUNDS.—In connection with the subject of Laparotomy for wounds of the intestines, it is generally understood that but three successful cases (for gunshot wounds) have been recorded in America: the first two by Dr. W. T. Bull, and the third one by Dr. J. B. Hamilton. It should be known that Dr. Bacon Saunders, of Bonham, Texas, has operated twice for wounds of the intestines, and once with success. The Doctor has performed abdominal section eleven (or twelve?) times, with only two deaths. This should be recorded, but the Doctor is too modest to assert his claims. We are promised, however, the report of the successful case alluded to above; and we want it to go on record with those of Bull and Hamilton; that Texas should have the credit of at least the fourth successful case.

---

THE next number of this JOURNAL will be printed in clean, new type throughout, and enlarged. Now is the time to subscribe.

---

DR. W. J. BURT.—It is proposed to issue in this JOURNAL, at an early date, an excellent engraved portrait of Dr. Burt, together with a biographical sketch. Our readers, we feel as-assured, will prize this as a *souvenir* of our departed friend and co-worker in the cause of medical science. Send in your subscriptions now to enable us to carry out this laudable desire.

---

A HEALTHY CITY.—Austin, the beautiful Capital, was never more healthy than at present, the scarlet fever having disappeared, and the dengue scare gotten up by *the* agitator has subsided.

---

We learn that scarlet fever, in a mild form, has appeared at Columbus, Texas.

---

We are in want of "Original Communications." Let us hear from you, subscribers.

---

Discounting the Future.—The following document was handed us by a gentleman from B—lank county, and is genuine. It is copied *verbatim et literatim:*

"To the Court of Blank County Tex this is to Certify that Mr Tom Pilere [Pillow.—Ed.] is not Abel to attend Court on the 9 of June written by Dr R. B. Blank Attenden Physician this the 5 day of June 86."

It will be observed that this "Attenden Physician," in addition to his professional attainments (which, it is to be hoped, are above his literary ditto) possesses the rare gift of divining the future, since he certifies "on the 5 day of June" that "Mr Pelere [Pillow] is not Abel to attend Court on the 9 of June." He does not leave room for the possibility of "Mr. Pilere's" getting better, or becoming "Abel." The certificate is a safe one, and the assertion therein cannot be gainsaid. A man cannot, in the nature of things, anticipate the future so far as to do a thing to-day which is to transpire at some subsequent period. If he could do so, how nice it would be, in our leisure, to dispose of a lot of things that are going to crowd us later, but which can come only with the future!—to bottle up business for the fall, like;—or contemplating the loss of sleep every night next week,—to anticipate and make up that loss by sleeping every night next week *now*. It is somewhat like the man who, having lost one night's sleep, put two mattrasses on the bed to compensate for it,—to get two nights sleep at once, as it were.

---

Editorial Secrets.—We reproduce the following from *Gaillard's Medical Journal*. "The subscriber cannot know how vastly important he is to the editor. In turn, he plays the part of confidant, critic, consoler, judge. Without him the editor's work in life would be absolutely valueless, Inspiration would cease; industry lapse into idleness; careful compilation would be but the rearing of a structure for the first idle wind to level to the ground, while editorial acumen would be as the flowers of the field, doomed to perish on the morrow. * * * * Many are the faults condoned by the good subscriber, and many the short-comings which he has overlooked; many the friendly hints which he gives to the editor, and the timely suggestions which he offers. May heaven bless him and multiply him!

But there is also the delinquent subscriber. What is he to the editor? Does he know that it is his hand which has loaded the editor's life with burdens heavy indeed, but which must be borne with a smile, and lifted with constant patience? Does he know that it is he who robs the editor of necessary sleep; who turns his sweetest pleasures into bitter regrets? * * * Does he know that while he deems himself "careless" and "procrastinating" only, he is helping to pile up a load of debt upon the constant friend who sends him at regular intervals the freshest and best results of his mental labor! Ah! delinquent subscriber, think not that thy faults are venial! Pass not over the modest monthly reminder which asks thee to "remit," with indifference. Upon thy promptitude hangs the happiness of one who contributes greatly to thy pleasure, thy knowledge, and thy usefulness. Upon thy "remembrance" depends the welfare of one who forgets thee, never. Be not thou the one to make him anxious and unhappy, but warm his heart and stimulate his courage by thy practical assistance.—*Extract from "An Editor's Note Book."*

---

*Egotism Extraordinary.*—"I cannot say whether it will be a success or not: [because] I shall not be there. You will see very few distinguished persons [like me] there," so wrote the anonymous correspondent of the *New York Medical Record.* For modesty it takes the confectionary. If somebody would stick a peacock's feather in the seat of this fellows trowsers doubtless he would strut himself to death and the fool-killer would be spared a small job, and there would be a vacancy in the immortal one hundred.

### "STOP MY PAPER."

The following from the *Winfield Courier* is respectfully submitted without comment: "In the history of all newspapers it is seen that there have been fools who get on their ear at odd spells and stop their paper. If these people were not over wise in their own conceit, they would put their finger in the river, pull it out and see what effect it had. The individual who vainly imagines that a paper cannot survive without his support, ought to go away from the city awhile. When his lordship returns, he will find that nine-tenths of his friends did not know that he had gone, and the other tenth didn't care a cent. We know and hope some of our patrons will find things in the *Courier* they cannot endorse. Even the good book hits a trifle hard sometimes. To get mad and burn your Bible would not mend matters nor hurt the publishers. The same with an editor. You may call him all sorts of ugly names, and the paper would still be out on time. And what is less to your credit, you would sneak around every night and borrow a copy of your neighbor. It is safest and best to keep your vest well pulled down and your subscription paid up, if you can't afford to pay it ahead. These are 'seeds of kindness' we desire to scatter everywhere."

PUBLISHERS will please take notice that all books sent for review or notice in this JOURNAL must come post or expressage paid.

RELIABLE INSURANCE AT LOWEST RATES to Physicians, is furnished by the Security Mutual Benefit Society of New York. It is claimed that insurance can be carried in this Company at $5 per $1,000; and that only three assessments a year are laid. The Company is well endorsed. See advertisement and send for circulars etc. Mention this Journal.

*An Apostate Westerner.*—The bait thrown to Whitaker by the shrewd Philadelphia organizers of "The" Association of What-d'ye call'ems—limited, was too tempting; it caught him, and now *he* "cannot attend or in any way participate in the International Medical Congress as now proposed to be organized!" What a mighty fish a frog is sometimes!

